# Development of a Possible General Magnitude System for Number and Space

**DOI:** 10.3389/fpsyg.2018.02221

**Published:** 2018-11-19

**Authors:** Karin Kucian, Ursina McCaskey, Michael von Aster, Ruth O’Gorman Tuura

**Affiliations:** ^1^Center for MR-Research, University Children’s Hospital Zurich, Zurich, Switzerland; ^2^Children’s Research Center, University Children’s Hospital Zurich, Zurich, Switzerland; ^3^Neuroscience Center Zurich, University of Zurich and ETH Zurich, Zurich, Switzerland; ^4^Clinic for Child and Adolescent Psychiatry, German Red Cross Hospital, Berlin, Germany; ^5^Zurich Center for Integrative Human Physiology, University of Zurich, Zurich, Switzerland

**Keywords:** number, space perception, ATOM, magnitude processing, development, angles, children

## Abstract

There is strong evidence for a link between numerical and spatial processing. However, whether this association is based on a common general magnitude system is far from conclusive and the impact of development is not yet known. Hence, the present study aimed to investigate the association between discrete non-symbolic number processing (comparison of dot arrays) and continuous spatial processing (comparison of angle sizes) in children between the third and sixth grade (*N* = 367). Present findings suggest that the processing of comparisons of number of dots or angle are related to each other, but with angle processing developing earlier and being more easily comparable than discrete number representations for children of this age range. Accordingly, results favor the existence of a more complex underlying magnitude system consisting of dissociated but closely interacting representations for continuous and discrete magnitudes.

## Introduction

### Differentiation Between Different Aspects of Number and Space Processing

A strong association between numbers and space has been reported over the last years of research. However, reported findings refer to different aspects of numbers and space. Therefore, it is very important to differentiate between various characteristics of numerical and spatial processing and their interrelation to gain further understanding and disentangle the complex number-space association. In this vein, Patro et al. (2014) proposed a more differentiated discussion of the number-space interaction since different numerical and spatial tasks target different underlying representations. According to their four level system of spatial-numerical associations, the authors suggest two categories with a non-directional number-space mapping: (1) cross-dimensional magnitude processing (number: cardinal, space: non-directional), and (2) association between spatial and numerical intervals (number: interval, space: non-directional). The other two categories refer to directional number-space mapping requiring spatial directionality in a sense that larger numbers are generally associated with the right side in Western cultures, while smaller numbers are associated with the left: (3) associations between cardinalities and spatial directions (number: cardinal, space: directional), and (4) associations between ordinalities and spatial direction (number: ordinal, space: directional). The present study focuses on cross-dimensional magnitude processing. This includes the examination of interrelations between cardinal aspects of non-symbolic numerosities (e.g., arrays of dots) and non-directional spatial dimension (e.g., line lengths, angles, sizes). Accordingly, when we talk about or discuss the number-space link in the present study, we exclusively refer to the above-mentioned numerical and spatial characteristics. In detail, number processing was explored by non-symbolic number comparison including two sets of dot clouds and spatial processing by comparison of two angles. Both tasks demand a magnitude judgment, which is based either on the evaluation of discrete quantity estimation of numerosity (number) or on continuous spatial processing (space).

### A General Cognitive Magnitude System

Associations between such different kinds of magnitude processing have led to the hypothesis of the existence of a shared general cognitive magnitude representation. Walsh (2003) proposed in “A Theory of Magnitude (ATOM)” that quantity, space, and time are part of a general magnitude system. Recent research has investigated to what extent and why these representational systems are shared. According to the content of the present study, we are mainly providing examples of cardinal numerical and non-directional spatial interactions. For an overview about associations between all dimensions (number, space, time, size, speed) according to ATOM see the review by Bueti and Walsh (2009).

Crucial contributions to the origin and existence of cross-dimensional magnitude processing stem from recent research in infants, brain imaging studies in adults, and single-cell recordings in primates or animals. Different studies highlight that a predisposition to relate numerical information to spatial magnitudes emerges very early in life (de Hevia and Spelke, 2010; Lourenco and Longo, 2010; de Hevia et al., 2012a,b). For instance, de Hevia and Spelke (2010) could show that infants as young as 8 months are sensitive to the association between non-symbolic numerical magnitudes and spatial line lengths. Moreover, also when continuous spatial variables are held constant, infants still attend to numerical change, indicating that number is spontaneously represented by young infants and both spatial and number information are probably integrated in an early magnitude representation (Brannon et al., 2006; Cordes and Brannon, 2009; Starr and Brannon, 2015). Finally, de Hevia et al. (2014) provided evidence that representations of space, time, and number are interrelated in even 0 to 3-day-old neonates.

Studies in adults corroborate a strong relation between number and space on both the behavioral and neuronal levels. Repeatedly a behavioral interference between the judgment of different magnitudes has been reported (Hurewitz et al., 2006; Longo and Lourenco, 2010; Dormal and Pesenti, 2013). On the neuronal level, several studies depicted an overlay of brain activation localized in the parietal lobes for different magnitudes (e.g., Fias et al., 2003; Dormal et al., 2012; for review see Pinel et al., 2004; Hubbard et al., 2005; Kaufmann et al., 2008). And particularly, the right intraparietal sulcus moved into focus as locus of a possible general magnitude system (for review see Sokolowski and Ansari, 2016). More recently, McCaskey et al. (2017) identified in adolescents the occipito-parietal stream as a common magnitude system for numerical and spatial magnitude comparisons assessed with the same task used in the present study.

Finally, animal behavior suggests that many animal species show a representation of space, number, and time (for review see Gallistel, 1989) and single-cell recordings in primates revealed identical neurons within the posterior parietal cortex that code for discrete non-symbolic numerosities (arrays of dots) and continuous spatial quantity (length) (Tudusciuc and Nieder, 2007).

Taken together, various sources of evidence suggest that number and space are processed by a general magnitude system that is claimed to develop very early in life and comprises identical brain areas of the parietal lobules. However, Bueti and Walsh (2009) emphasize in their latest review that although the parietal lobe may be considered as the “primary magnitude cortex,” it is only one locus of magnitude processing and that there is a magnitude system and not a single magnitude area. Therefore, it is also not surprising that only some activation sites for number, space, and time overlap and a few do not. Furthermore, Bueti and Walsh (2009) point out that an over simplistic view of a general magnitude system would assume systematic interferences between number, space, time and all kinds of magnitudes. This is clearly not the case. In fact, Dormal and Pesenti (2007) reported only an interference effect of space with numbers, whereas Nys and Content (2012) showed the reciprocal interference. Moreover, Hurewitz et al. (2006) demonstrated interference between discrete and continuous stimulus dimensions in both directions. Not only are reported findings inconsistent about the directions of interferences between different magnitudes, Agrillo et al. (2013) and Barth (2008) found absolutely no correlations among non-symbolic estimations (number/space/time or number/space) contradicting the existence of a general magnitude system. Similarly, behavioral and neuronal findings from Cappelletti et al. (2014) also point to distinct systems for quantifying different magnitudes. Their results showed that the proficiency in numerical and continuous quantity tasks was not correlated in participants with a specific math learning disorder (dyscalculia) (e.g., impaired number but spared time and space processing), and moreover, performance in these tasks was partly dissociated in subjects without math problems, both behaviorally and anatomically. Similar findings from populations with specific impairments in one quantitative domain reported preserved abilities in other magnitude domains (Mussolin et al., 2011; Rousselle et al., 2013; Crollen and Noël, 2015). Lourenco et al. (2012) also reported only partly overlapping representations of numerical and spatial magnitudes by showing that number and spatial performance correlated with higher mathematical competence, but number precision contributed uniquely to advanced arithmetic and spatial precision uniquely to geometry in adult subjects. Similar work in children by Lourenco and Bonny (2017), however, revealed no differentiation between number and spatial performance – the precision of both tasks contributed exactly to the same math measures (calculation and geometry). On the other hand, there is also evidence speaking for a correlation between number and spatial processing, as expected under a general magnitude system. Lourenco and colleagues revealed positive correlation between the performance of comparisons between non-symbolic number arrays and cumulative area in typically developing children (Lourenco and Bonny, 2017) and adults (Lourenco et al., 2012).

Results from DeWind and Brannon (2012) are contradictory to ATOM, which predicts that improving abilities in one domain (e.g., number) would improve other quantitative domains (e.g., space). In this regard, DeWind and Brannon (2012) administered a simple numerical training, reporting an improvement in numerical skills but not in a spatial task. Due to this lacking transfer effects, training only one domain and hoping for improvements in the untrained domain makes no sense. However, interventions focusing on the improvement of the association between number and space are supposed to be more beneficial for basic geometrical and numerical understanding (reviewed by Cipora et al., 2015; Hawes et al., 2017).

In sum, there is no doubt about a strong connection between number and space, however, if both representation originate from a single general magnitude system is contradictious and further research is needed.

### Development

An important determinant in the explanation of different findings could be characteristics of investigated populations such as age. Regarding development, findings suggest that we are born with the ability to relate numerical and spatial factors (de Hevia et al., 2014), which probably get further integrated over development as can be observed by directional biases in spatial or numerical line bisection tasks in younger children (7 years of age) to an adult-like behavior in 13-year-old children (van Vugt et al., 2000; Hausmann et al., 2003; Göksun et al., 2013). Hence, it can be inferred from these findings that school-age might be still a critical period in the development of numerical and spatial skills. However, only very little knowledge is available today at this age-range. To our knowledge, only one study examined the relation between spatial and numerical skills over development in school aged children and concluded rather differing mechanisms underlying physical and numerical space in childhood that might integrate in adulthood (Göksun et al., 2013).

Speaking about development, it has to be kept in mind that not only the mere existence of a general magnitude system is disputable, but also different possible developmental trajectories are currently discussed (for review please see Lourenco and Longo, 2011; Lourenco, 2015). According to the classic approach of learning by Gibson and Gibson (1955), the *differentiation view* suggests strongest cross-dimensional associations earlier in life and an increase in differentiation of representations of magnitude dimensions over development. In line with this differentiation view, Newcombe et al. (2015) come to the conclusion in their review that infants begin with a general magnitude system which differentiates into distinct dimensions over developmental time. In contrast, the *enrichment view* assumes an increase in strength of different magnitude representations over development.

### Aim of the Present Study

The goal of the present study was to examine the relation between discrete non-symbolic number processing (arrays of dots) and continuous spatial magnitude processing (angles) taking the important aspect of development into consideration. Therefore, we investigated typically achieving children spanning different school grade levels. Izard and Spelke (2009) have shown that sensitivity to detect relationships of line length and angles shows steady improvement over childhood, reaching asymptote at about 12 years of age. However, the authors also reported differences in the developmental trajectories of length and angle sensitivity, while the sensitivity to length is mature by the age of 8, sensitivity to angle continues to mature until 10. In addition, and as mentioned above, adult-like behavior has been observed in 13-year-old children in spatial or numerical line bisection tasks. Accordingly, the current work focusses on children between 8 and 13 years, as this age range seems to be an interesting developmental stage to test higher cognitive processing of angle and dot comparison. According to Walsh (2003) an interference between both tasks would support ATOM. Regarding development, we expect improvements in numerical and spatial quantitative skills. As the development of numerical and spatial representation is a complex process, different developmental trajectories are possible. The investigation of these developmental courses could provide further evidence for the existence of a general magnitude system or for separate cognitive representations for discrete and continuous magnitudes. On the one hand, a strong cross-dimensional transfer in earlier grade levels and/or parallel development for number and space abilities would support ATOM (proposed by Walsh, 2003). On the other hand, increasing integration among numerical and spatial magnitudes over development and/or dissociated developmental pathways would rather support the idea that quantitative thinking begins with the ability to discriminate between continuous properties. Over development, children learn the correlation between continuous and discrete features suggesting that discrete and continuous magnitude processing are two separate, but interacting systems underlying a general magnitude system (proposed by Leibovich and Henik, 2013a).

To address these hypotheses we decided to test non-symbolic number processing by the comparison of number of dots and spatial magnitude processing by a clearly different stimulus type, namely angle size. This is in contrast to some studies that use exactly the same arrays of dots for both dimensions by asking two different questions: which of two arrays is greater in number (numerical estimation) or cumulative area (spatial estimation). Although such a design has the advantage of using exactly the same stimuli for the two tasks, it has the disadvantage that participants have always to keep in mind which question they have to answer at the moment and even more importantly, they have to inhibit the processing of the irrelevant dimension. Both additional mental processes are not of interest in our study and put a supplementary challenge especially for children. Finally, research with infants proved that they are already able to discriminate 2-dimensional angles (Slater et al., 1991) and findings from preschool children corroborated generally high performance levels of angle comparisons and provide evidence that the dimension of angle is even more salient than length for children (Izard and Spelke, 2009). Therefore, the present study design testing children’s magnitude processing skills uses dot array versus angles.

## Materials and Methods

### Subjects

In total 369 children participated in the present study, of which 2 were excluded due to incomplete task performance, resulting in a group size of 367 children between 8.2 and 12.9 years of age (*M* = 10.6; *SD* = 1.1), including 39% girls and 61% boys. Children attended third to sixth school grades, such that 87 children were in the third grade (8.2–10.2 years of age: *M* = 9.3; *SD* = 0.4), 140 in the fourth grade (9.3–11.8 years of age: *M* = 10.3; *SD* = 0.4), 110 in the fifth grade (10.1–12.7 years of age: *M* = 11.4; *SD* = 0.5), and 30 in the sixth grade (11.0–12.9 years of age: *M* = 12.3; *SD* = 0.4).

The study was approved by the local ethics committee (Kantonale Ethikkommission Zürich) based on guidelines from the World Medical Association’s Declaration of Helsinki (WMA, 2002). According to the local ethical committee, written parental consent was not required as no risk for the children existed, voluntariness and privacy was guaranteed at all times. Data collection was fully anonymised and took place in the scope of a lecture of the Children’s University of Zurich to illustrate our research field, research question, and research experiments. Children’s University of Zurich gave also their consent to analyze obtained data.

### Non-symbolic Number Comparison Task

**FIGURE 1:**
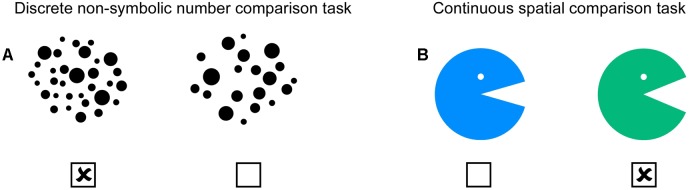
Numerical and spatial task. **(A)** In the non-symbolic number comparison task, children were asked to indicate which set includes more dots. **(B)** In the spatial comparison task, children had to mark the Pacman with the bigger mouth. Both tasks consisted of 28 different pairs, each of which were controlled for factors not of interest. Children had 2 min time for each task to tick the correct solutions. All trials of a task were printed on a double-sided A4 page.

Non-symbolic number comparison performance was tested with a paper-and-pencil task including a total number of 28 different trials (see Figure 1A). In each trial two groups of dots including a range from a minimum of 8 to a maximum of 32 dots were presented horizontally. Children were asked to indicate on which side more black dots were presented. Presentation of dots was controlled for individual size of dots (no judgment possible due to individual dot size), total displayed area (no judgment possible due to total black area), distribution of dots (no judgment possible due to total covered area), the total number of presented dots for each numerical distance between sets (control for size effect), the side of correct answer, and comparable number of trials for each numerical distance between presented magnitudes were presented (distance 2 = 4 trials, distance 4 = 4 trials, distance 6 = 6 trials, distance 8 = 5 trials, distance 10 = 5 trials, distance 12 = 4 trials). Ratio between smaller and larger dot arrays was 0.4, 0.5, 0.6, 0.63, 0.67, 0.70, 0.71, 0.77, 0.8, 0.83, 0.9, or 0.91. Detailed information about all 28 trials can be found in the Supplementary Table S1. All children were carefully introduced to the task and encouraged to solve all trials by comparison of both sets of presented dots by numerical estimation and not counting. To further prevent children from counting, time was restricted to 2 min for all 28 trials. The ability of non-symbolic magnitude comparison by dots requires a decision about discrete quantity.

### Spatial Comparison Task

In the spatial comparison task, a green and a blue Pacman facing to the right side with varying mouth size was presented horizontally (see Figure 1B). Children had to indicate by pencil which of the two presented Pacmen has a bigger mouth, whereas line length intersecting the angle was controlled and corresponded always to radius of the circle. In contrast to the non-symbolic number comparison task, this task requires a visuo-spatial and continuous magnitude decision. The mouth angle of one Pacman was always 45 degrees and the mouth angle of the other Pacman varied between minimum 18 degrees to maximum 72 degrees [18, 23, 27, 32, 36, 40, 42, 47, 49, 54, 59, 63, 68, 72 degrees (2 trials for each degree)]. Difficulty level was controlled by varying the ratio between both presented mouth angels across all trials. Detailed information about all 28 trials can be found in the Supplementary Table S2. In addition, the side of the correct answer and color of Pacman were balanced. Similar to the number comparison task, children were carefully instructed and advised to solve the spatial comparison task by simple estimation of mouth sizes and not to use for instance their fingers or any other tool to measure the mouth sizes. Again, children had 2 min time to solve all 28 trials.

### Data Analyses

For both tasks, the non-symbolic number and the spatial comparison task, the percentage of correctly solved trials was calculated. Subsequent statistical analyses were performed with IBM SPSS Statistics Version 22. As accuracy levels of number and spatial comparisons were negatively skewed and the assumption of normality was therefore violated, non-parametric tests were used. First, we were interested to see which task is more difficult. Therefore, the percentage of correctly solved trials between both tasks was compared by the Wilcoxon signed-rank test. Subsequently, *post hoc* Wilcoxon signed-rank comparisons between both tasks were performed for each grade level individually. Second, to test if numerical and spatial processing are related, Spearman’s correlation coefficients were calculated between both tasks over all grade levels and for each grade level individually. Third, development across grade levels was evaluated by Kruskal–Wallis test, and the *post hoc* Mann–Whitney test was conducted to test for developmental differences between grade levels. Finally, effect sizes are reported for all major findings with the denomination *r* for dependent Wilcoxon tests and Spearman’s correlations, and the denomination *q* that permits to interpret the difference between two correlations.

## Results

All 367 children were able to solve all 28 trials of both tasks within the allotted time of 2 min for each condition and performed clearly above chance level. The median accuracy for the non-symbolic number task ranged from 61–100% across grade levels (third grade Mdn = 92.9 (IQR 89.3–96.4); fourth grade Mdn = 96.4 (IQR 92.9–96.4); fifth grade Mdn = 96.4 (IQR 92.9–100); sixth grade Mdn = 96.4 (IQR 92.9–100). Similarly, the median accuracy for the spatial comparison task ranged from 57.1–100% (third grade Mdn = 92.9 (IQR 89.3–96.4); fourth grade Mdn = 92.9 (IQR 89.3–96.4); fifth grade Mdn = 92.9 (IQR 89.3–96.4); sixth grade Mdn = 96.4 (IQR 92.9–100). Please see Figure 4.

**FIGURE 2:**
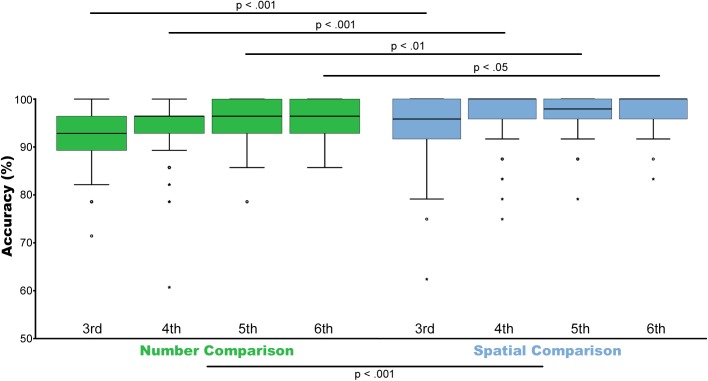
Accuracy. Illustrated are median, interquartile range (IQR = length of box) and lowest and highest values which are no greater than 1.5 times the IQR (whiskers) of percentage correctly solved trials for non-symbolic number comparison (green) and spatial comparison (blue) from the third to the sixth grade. Outliers are marked by circles (1.5–3 times the IQR from the quartile) or asterisks (a value >3 times the IQR from the quartile). Wilcoxon test showed that spatial comparison is in general significantly easier compared to non-symbolic number comparison (*p* < 0.001). Analyses between individual grades indicated difference between the number and spatial task in the third (*p* < 0.001), fourth (*p* < 0.001), fifth grade (*p* < 0.01), and sixth (*p* < 0.05) grade. Only trials with matched ratios between conditions were included.

For any statistical comparisons between both magnitude dimensions, only identical ratios were included in the analyses to prevent any confounding effects due to subtle differences in ratios between tasks. Examining only trials with matched rations in both conditions resulted in 25 different trials for the number task and 24 trials for the space task. Ratios included in this balanced analysis were as follows: 0.4/0.5–0.51/0.6/0.63/0.66–0.67/0.71/0.76–0.077/0.8/0.83/0.89–0.9/0.91–0.92. Please see Supplementary Table S3 for detailed information. Including only matched ratios, the median accuracy for the non-symbolic number task ranged from 60–100% across grade levels [third grade Mdn = 92 (IQR 88–96); fourth grade Mdn = 96 (IQR 92–96); fifth grade Mdn = 96 (IQR 92–100); sixth grade Mdn = 96 (IQR 92–100)]. Similarly, the median accuracy for the spatial comparison task ranged from 63–100% [third grade Mdn = 95.8 (IQR 91.7–100); fourth grade Mdn = 100 (IQR 95.8–100); fifth grade Mdn = 97.9 (IQR 95.8–100); sixth grade Mdn = 100 (IQR 95.8–100)]. Please see Figure 2.

### Number or Space Comparison: Which Task Is More Difficult?

Results of the Wilcoxon test for identical ratios revealed that spatial comparison (Mdn = 100) is generally easier (*z* = −6.771, *p* < 0.001, *r* = −0.25, *N* = 366) compared to non-symbolic number comparison (Mdn = 96). Analyses between tasks for different grade levels individually revealed significant difference between the number and spatial task in the third *z* = −3.534, *p* < 0.001, *r* = −0.27, *N* = 87; fourth grade *z* = −4.940, *p* < 0.001, *r* = −0.29, *N* = 139; fifth grade *z* = −2.7, *p* < 0.01, *r* = −0.18, *N* = 110; and sixth grade *z* = −2.083, *p* < 0.05, *r* = −0.27, *N* = 30. Please see Figure 2.

**FIGURE 3:**
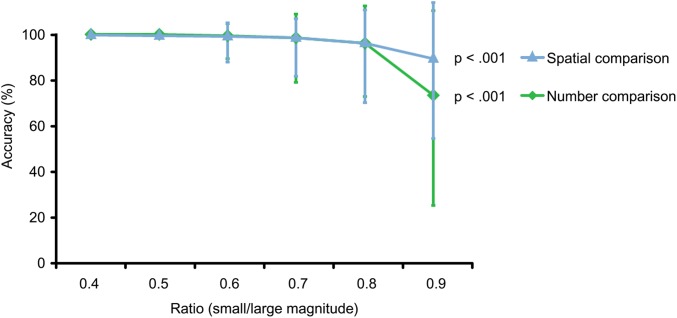
Ratio effect. With increasing ratio between magnitudes, task difficulty increases for both tasks, which is reflected in decreasing accuracy levels for spatial comparison (blue) *p* < 0.001 and number comparison (green) *p* < 0.001. Illustrated are medians and interquartile ranges for each ratio. Only trials with matched ratios between conditions were included.

In addition Figure 3 illustrates that accuracy levels decreased significantly for both conditions with increasing ratio between magnitudes, whereas bigger ratios stand for smaller distances between magnitudes and are therefore more difficult to compare (Spearman’s correlation for number comparison: *r*_s_ = −0.961, *N* = 25, *p* < 0.001; and for spatial comparison: (*r*_s_ = −0.880, *N* = 24, *p* < 0.001). Differences between conditions for different ratios did not reach significance. Please see Figure 3.

### Are Non-symbolic Number and Spatial Abilities Related?

Spearman’s correlation over all grade levels showed that the accuracy for both tasks with matched ratios are significantly and positively related with each other *r*_s_ = 0.264, *N* = 366, *p* < 0.001, also when partialling out age (*r* = 0.257, *N* = 363, *p* < 0.001) or grade level (*r* = 0.250, *N* = 363, *p* < 0.001) or age and grade together (*r* = 0.247, *N* = 362, *p* < 0.001). *Post hoc* analyses within grade levels supported a relation between both magnitude dimensions. Significant and positive correlations between the number and spatial task were also found within third, fourth, and sixth grade (third grade *r*_s_ = 0.295, *N* = 87, *p* < 0.01; fourth grade *r*_s_ = 0.305, *N* = 139, *p* < 0.001; sixth grade *r*_s_ = 0.386, *N* = 30, *p* < 0.05), but not within fifth (fifth grade *r*_s_ = 0.124, *N* = 110, *p* = 0.196).

Further, we were interested to evaluate if the strength of correlation between both tasks decreases with development, as the analyses of correlations between both tasks for each grade level pointed into this direction. Therefore, we performed comparison of correlation coefficients between grade levels, using Fisher r-to-z transformation. This revealed significant differences between correlation coefficients of number and space between third and fifth grade (one-tailed *p* < 0.05, Cohen’s *q* = 0.267) and between fourth and fifth grade (one-tailed *p* < 0.01, Cohen’s *q* = 0.337), pointing to a weaker correlation between magnitude dimensions in fifth grade compared to lower grades. Comparisons between the strength of correlations between number and space of the sixth grade and lower grades turned out not to reach significance.

### Development of Non-symbolic Number and Spatial Skills

**FIGURE 4:**
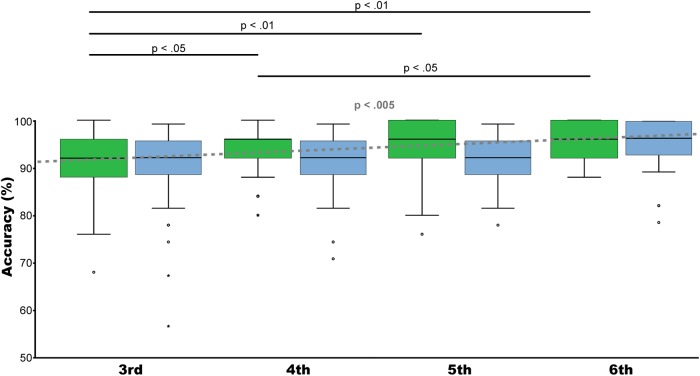
Development. Illustrated are median, interquartile range (IQR = length of box) and lowest and highest values which are no greater than 1.5 times the IQR (whiskers) of percentage correctly solved trials for non-symbolic number comparison (green) and spatial comparison (blue) from the 3rd to the 6th grade. Kruskal–Wallis test showed an increase in mean accuracy over grade levels only for non-symbolic number comparison (*p* < 0.005) (gray dotted line). *Post hoc* analyses revealed significant performance differences between 3rd and 4th (*p* < 0.05), 3rd and 5th (*p* < 0.01). Third and 6th (*p* < 0.01) grade and between 4th and 6th grade (*p* < 0.05). Trials of all ratios were included.

The developmental course from the third to the sixth grade level of both tasks was evaluated by Kruskal–Wallis test including the performance of all ratios of the two tasks (non-symbolic number task and spatial comparison task) as dependent variable and grade level as independent variable. Results indicated that only for non-symbolic number comparison a significant developmental effect over grade levels could be observed [*H*(3) = 15.688, *p* < 0.005], but not for spatial comparison performance [*H*(3) = 6.848, *p* = 0.77]. *Post hoc* Mann–Whitney test comparison for non-symbolic number comparison performance showed a significant difference between third and fourth (*U* = −1.980, *p* < 0.05), third and fifth (*U* = −3.235, *p* < 0.01), third and sixth (*U* = −3.364, *p* < 0.01), and between fourth and sixth (*U* = −2.079, *p* < 0.05) grade levels. Please see Figure 4.

Comparable developmental effects were found when calculating Spearman’s correlations between task performance, age, and grade level. Only non-symbolic number comparison correlated significantly with age (*r*_s_ = 0.157, *N* = 367, *p* < 0.01) and grade level (*r*_s_ = 0.205, *N* = 367, *p* < 0.001). Spatial comparison did not reach significance with age (*r*_s_ = 0.034, *N* = 364, *p* = 0.514), or grade level (*r*_s_ = 0.063, *N* = 364, *p* = 0.229).

## Discussion

The present study aimed to further elucidate the association between number and space, which has been proposed to rely on a common general magnitude system (Walsh, 2003; Bueti and Walsh, 2009). However, conflicting research findings called into question whether processing of different dimensions of magnitudes can be attributable to such a general magnitude system. To extend the current body of literature, we investigated the relationship between discrete non-symbolic number processing and continuous spatial magnitude encoding, taking the impact of development into consideration. To our knowledge, this represents the first attempt to investigate a developmental association between these quantity skills in children between the third and sixth grade. Discrete non-symbolic number processing was tested by means of a comparison task of arrays of dots and continuous spatial processing by the comparison of angle sizes.

In sum, results indicated that angle comparisons were generally easier compared to non-symbolic numerical comparisons for children between the third and sixth grade. Moreover, the larger the ratio between magnitudes that had to be compared the more difficult both conditions became. Second, both tasks were significantly related with each other over the entire examined age range, also when controlling for age and/or grade level. However, third, and lastly, our findings suggest differences in the developmental course of discrete and continuous magnitude processing: significant improvements of discrete numerical processing from the third to the sixth grade can be found, whereas continuous spatial representation might have already reached ceiling levels at this age range.

### Number or Space Comparison: Which Task Is More Difficult?

Overall, both tasks got more difficult as the ratio between the two magnitudes increased. This is consistent with the well described distance and size effects which are characterized by increasing difficulty with smaller numerical distances and the larger total numbers of dots to be compared (Moyer and Landauer, 1967). Both effects can be explained by the assumption that our representation of quantitative dimensions become increasingly imprecise and noisy with increasing magnitudes (Feigenson et al., 2004). This seems to be true for both, discrete non-symbolic magnitude and angle processing.

It is important to take into account that ratios between smaller and larger magnitudes differed slightly between both conditions. This might lead to difficulty differences between tasks due to task design in favor to the non-symbolic number comparison task. Therefore, we included only trials with identical ratios of both conditions in order to compare accuracy levels between conditions. Results showed that for matched ratios between tasks spatial judgment of angle size is easier compared to non-symbolic magnitude comparison. This result is consistent with findings from Leibovich and Henik (2013a), who also showed higher accuracy levels for a continuous spatial task compared to non-symbolic dot comparison. They hypothesize that the superiority of processing continuous magnitudes, together with the fact that evolutionary ancient species such as fish and bees are also able to process continuous magnitudes, might indicate that the system for continuous magnitudes is older than the system for processing discrete magnitudes. Our data can lend support to this assumption that the system for continuous quantity might develop earlier during childhood than the discrete quantity system. In addition, present findings are in line with results by Odic et al. (2013), who also showed higher acuity for area representation than number representation in 3- to 6-year-old children by comparison of discrete non-symbolic number processing (comparison of dot arrays) and continuous spatial processing (comparison of area sizes).

In addition, both tasks were constructed in a way that confounding factors, such as visual cues, could be excluded to a large degree since many studies have shown that especially in non-symbolic dot comparison tasks results could be biased by visual perceptual cues (e.g., Gebuis and Gevers, 2011; Cleland and Bull, 2015).

Taking these considerations into account, the present findings demonstrate that continuous spatial judgments seems to be easier for school children between third and sixth grade than non-symbolic number discrimination.

Finally, consideration should be given to general angle perception. In the present study, we have assumed that spatial processing of continuous angles is similar to other types of continuous spatial functions, whereas comparison between two angles is getting more difficult the closer both angles are (distance effect) and is getting more difficult with increasing angle sizes for a given distance between angles (size effect). In line with the distance effect, angle comparison got more difficult as the ratio between the two angles increased. However, future studies should test size effects in angle perception particularly.

### Are Non-symbolic Number and Spatial Abilities Related?

Present findings reveal that non-symbolic number processing is positively related to continuous spatial abilities in school children. However, since performance of number comparison increased significantly over age or grade levels this relation might have been driven rather by developmental processes. This possibility could be excluded by controlling the effects of age and/or grade level and additionally, correlations between both tasks were also found for third, fourth, and sixth grade level separately. This is in line with behavioral reports of significant interference between numerical and spatial processing in adults (Hurewitz et al., 2006; Longo and Lourenco, 2010; Dormal and Pesenti, 2013). In particular, both Hurewitz et al. (2006) and Dormal and Pesenti (2013) also reported a link between non-symbolic number comparison and continuous spatial processing in adults. Although research in childhood provides strong evidence of mapping numbers and space on a mental number line with a particular scaling (e.g., Siegler and Opfer, 2003; Siegler and Booth, 2004; Booth and Siegler, 2008; Moeller et al., 2009; Kucian et al., 2011) and direction (e.g., Patro and Haman, 2012; Hoffmann et al., 2013; Ebersbach et al., 2014), very little and critically discussed knowledge about children’s representation of symbolic numerosities and continuous space is available (de Hevia and Spelke, 2009; de Hevia, 2011; Gebuis and Gevers, 2011; Göksun et al., 2013; Odic et al., 2013; Cleland and Bull, 2015). To our knowledge, no study in terms of discrete non-symbolic numerical quantities in respect to continuous space mapping in school aged children exists to date. Therefore, the present findings could extend the current limited body of literature in school children, showing an interrelation between cardinal aspects of non-symbolic numerosities and non-directional spatial dimension processing. In contrast, Odic et al. (2013) could not find a significant correlation between number and area acuity in their sample of children once age was controlled. Hence, their results favor rather separate representations of number and area. Contrasting findings might be due to age differences, since children in Odic et al. (2013) study were much younger (3–6 years) compared to the present study (8–13 years). However, it has to be mentioned that reported findings in younger children are mixed and for instance Lourenco and Aulet (2018) reported in a recent study cross-magnitude interactions in infancy and at 3.7 years of age.

Comparison of correlation strength between different grade levels pointed rather to weaker relation between numerical and spatial representations in the fifth grade compared to lower grades. This lower strength of cross-dimensional correlation might hint to an increasing differentiation among magnitude dimensions from third to fifth grade, favoring the differentiation view of development. Similarly, Lourenco et al. (2012) reported a differentiated relation of numerical and spatial magnitude processing on arithmetic and geometry in adulthood, whereas no such differentiation was found in children (Lourenco and Bonny, 2017). However, in the present study, the tendency from third to fifth grade could not be extrapolated into the sixth grade. The comparison of correlation strength of sixth graders with lower grades reached not significance. This might be an effect of the smaller sample size in the sixth grade (*N* = 30). Correlations calculated on data collected from a small sample (30 or fewer subjects) can be affected substantially by dissimilar distribution shapes (Goodwin and Leech, 2006). Whereas in larger sample sizes, there is no direct bearing of sample size to the size of the correlation coefficient (Goodwin and Goodwin, 1999). Accordingly, it might be possible that the examination of a larger sample in the sixth grade would lead to significant differences in correlation strength between sixth and lower grades. However, we cannot tell if correlation strength in sixth grade would be weaker or stronger compared to lower grades. Fact is, that we found no differences between sixth grade and lower grades in the present study and therefore when we consider the total examined age range from third to sixth grade, present data does not legitimate a conclusion in favor of the differentiation or the enrichment view. Future studies should use identical stimuli, as in the present work, in broader age ranges with comparable sample sizes to test the hypothesis of differentiation between magnitude dimensions, because it might also be possible that decreased correlation between dimensions is due to increased general task performance over development.

### Development of Non-symbolic Number and Spatial Skills

Various attempts have been made to investigate the development of non-symbolic number processing, but far fewer have examined the development of continuous spatial skills in children. The present study allows not only insights into the developmental course of both skills to be drawn, but also lends insight into the relationship between them.

Regarding number development, our results are in line with existing knowledge showing that children became more accurate when two non-symbolic magnitudes have to be compared with increasing grade level or age. The nature of children’s non-symbolic magnitude representation is thought to index the precision to process numerical quantity information in an approximate way (Dehaene et al., 1999; De Smedt et al., 2013). Halberda and Feigenson (2008) showed that the resolution of this system continues to increase throughout childhood – children perform more accurately and faster on magnitude comparison tasks with increased precise representation.

However, it has also to be taken into account that non-symbolic magnitude comparison tasks in an experimental design as used in the present and other scientific studies control for as many visual nuisance factors as possible to prevent that subjects are able to base their magnitude judgment not on number, but on other magnitude dimensions such as spatial extent. At the same time, such a controlled presentation of non-symbolic magnitudes does usually not correspond to natural surroundings, where more apples take up more space. Therefore, it needs cognitive demands to suppress irrelevant magnitude dimensions in a controlled experimental setting and hence, increased performance in non-symbolic discrete magnitude comparison might also be explained by increased abilities of children in these rather domain-general cognitive capacities than pure numerical abilities (Szucs et al., 2013).

In contrast to numerical magnitude judgment, our findings suggest that from third to sixth grade, children seem not to improve in continuous spatial processing, which is indicated by no correlation between spatial performance levels and age or grade level. Accordingly our data rather indicate no improvements over developmental time in the capacity to compare continuous spatial dimensions at this age range. Alternatively, these data might also be interpreted in a way that discrete numerical magnitude representation is still developing from the third to the sixth grade, whereas continuous spatial processing already reached ceiling level in this age range. In line, Odic et al. (2013) reported similar growth pattern across development for number and area processing in preschool children, but with improvements in area acuity occurring more quickly than in number acuity. The authors argue that these results suggest both an underlying similarity and an important difference between discrete non-symbolic number processing and continuous spatial processing.

### General Magnitude System

At large, the present study aimed to gain knowledge about the relation between discrete non-symbolic number encoding and continuous spatial magnitude processing accounting for developmental effects. To date, research has revealed a largely inconclusive picture with respect to an underlying common magnitude system to process both quantity dimensions.

Regarding ATOM, it has been proposed that children with difficulties in one quantitative domain, e.g., numerical processing, should have difficulties in all magnitude domains, e.g., spatial and temporal encoding. Applied to our study, a child with problems in the number task should also be weak in the spatial task, resulting in equal performance levels between both quantitative tasks. However, in our point of view the mere difference in accuracy levels is a very weak indicator of the relation between two tasks and does not justify the support or contradiction of ATOM. Moreover, it is possible that a single processing system is more prone to one or the other input modality, e.g., due to familiarity, leading to performance differences. In the same vein, acuity of a given magnitude depends on the format of the stimuli, and differences in accuracy levels between different stimuli types, as used in the present study, are probably driven by the stimuli type and not explicable by different magnitude representations (Price et al., 2012; Gilmore et al., 2015). In this sense, interferences such as transfer effects of training one competence on another (as has been carried out for instance by DeWind and Brannon, 2012), priming effects or correlative analyses between tasks are more meaningful.

In the present study, correlation analyses between both tasks pointed to a relation between number and space processing. This link was independent of age or grade level, as the correlation between number and space was still significant when controlling for both factors. Accordingly, we can conclude that discrete non-symbolic number processing and continuous spatial processing are related in school-aged children, but if both skills are processed by a single magnitude system or by two closely interacting systems remains unclear. However, when we take observed differences in the developmental courses of number and space processing into account, the present study provides stronger evidence for two dissociated, but closely related magnitude systems.

On the grounds of current literature and present findings, the description of ATOM as initially proposed by Walsh (2003) seems to be over simplistic as also pointed out by Bueti and Walsh (2009) themselves. Present findings favor suggested models by Leibovich and colleagues (Leibovich and Henik, 2013a,b; Leibovich et al., 2017), who postulate that we are born with the ability to discriminate between continuous properties. As a matter of fact, continuous and discrete properties of arrays of dots for instance are inseparably linked (for review see Leibovich and Henik, 2013b). This is also the case in the present study. Although we have tried to control as many visual confounds as possible in the non-symbolic magnitude comparison task, such as the total surface area of the dots, size of dots, their density, etc., the arrays always contain continuous properties as well. Non-symbolic number comparisons always carry continuous properties that are correlated with numerosities and a separation is physically not possible. Consequently, over development we learn the correlation between continuous and discrete features, which allows us to use both properties to estimate magnitudes. In line with their assumption, our results point to developmental differences of continuous spatial and discrete non-symbolic number processing, with continuous representation being sufficiently developed in third grade children. In contrast, discrete number estimation is still developing and generally more demanding for school children. Moreover, Leibovich and Henik (2013a) suggest that discrete and continuous magnitude processing are two separate, yet interacting systems underlying a general magnitude system (see also Leibovich et al., 2013). Similarly, current findings showed a link between both number and space processing, also when controlling for age and/or grade level effects, supporting an interaction between systems. On the other hand, differences in general performance levels and developmental trajectories found in the present study also point to partly independent systems. Such a complex interrelated representation of space and number might also explain why the ability to create number–space connections provides only limited links to mathematical learning (reviewed by Cipora et al., 2015). Moreover, Lourenco and Longo (2011) emphasized that characterizing the development of a general magnitude system is complicated and developmental accounts, which consider only differentiation or integration of different magnitudes over time are likely to be incomplete.

Finally, as we have emphasized in the introduction section, it is very important to differentiate between various characteristics of numerical and spatial processing and their interrelation to gain further understanding and disentangle the complex number–space association. In particular, many studies examine the comparison of dot arrays (as in the present study) with area, total cumulative area or line length. In this sense, the present findings add further knowledge on another dimension of continuous spatial processing, namely angles. Accordingly, differences in stimuli type should be considered in the interpretation of different findings. Future research is needed to particularly investigate the relation of discrete non-symbolic number comparison with a variety of continuous types of spatial judgments (area, total cumulative area, angle, length, etc.) to gain a differentiated picture about their relations over development and a possible underlying general magnitude system.

### Limitations

As mentioned earlier, present findings are not able to explain the principle of a possible general magnitude system conclusively and some limitations have to be considered. First, although there is lots of evidence showing a relation between different magnitude dimensions, which has been argued to originate from a common general magnitude system, also other explanations for such a crosstalk are possible. Van Opstal and Verguts (2013) for instance propose instead of a general magnitude system that different magnitude representations are processed separately, but share a decision/response procedure or working memory demands which lead to observed similarities between different magnitude dimensions. Similarly, we are not able to distinguish if errors in either task are based on difficulties in number and/or spatial processing or are rather a result of diminished executive functions, like reduced attentional or inhibitory control. However, as our task required no working memory, a relation between dimensions based on common working memory procedures can be excluded. Moreover, the expected and observed increase in difficulty with increasing ratio between sets also speaks against effects of general decision/response procedures or differences in executive processes. Nevertheless, future studies examining numerical and angle processing with a task (e.g., habituation task or priming task) that is not dependent on domain-general functions and does not require a decision or a response would provide more information regarding this debate. For the relation between non-symbolic numerosity and total cumulative area, Lourenco et al. (2016) tested transfer effects across magnitudes in a subliminal priming paradigm. Their findings suggest that number and area are not fully differentiated, as primed numerals had an effect on performance of cumulative area judgments.

Second, it has to be noted that the present study served as survey of children’s non-symbolic numerical and spatial magnitude discrimination abilities to develop a sophisticated paradigm examining also underlying neuronal processes (McCaskey et al., 2017). This is the reason why continuous ratios for numerical and spatial comparisons were chosen to map children’s performance levels as thoroughly as possible, but included also slightly different ratios between dimensions. Therefore, it is mandatory to include only identical ratios of number and space judgments as soon as you do any comparison between both magnitude dimensions. Accordingly, we performed separate analyses, including only matched ratios between both tasks to draw clear-cut conclusions regarding comparisons between magnitude dimensions. Correspondingly, Figure 2 including only matched ratios illustrates higher accuracy levels for spatial comparisons. In contrast, when including all ratios this effect seemed to be reversed, please see Figure 4. However, this is falsified by the fact that the spatial comparison task included more trials with higher ratios, which are more difficult to be compared. Therefore, it is important to compare difficulties between conditions only for identical ratios.

Third, performance levels were generally high, why possible ceiling effects have to be considered. However, non-parametric statistical analyses revealed significant differences between both tasks, even when controlling for age or grade level effects, corroborated that difficulty increases with smaller distances between magnitudes that had to be compared, and finally showed improved performances from third to sixth grade for number comparison. None of these effects would be expected if strong ceiling effects were present. However, decreased strength of correlation between number and space from third to fifth grade might be explained by increased general performance up to ceiling levels.

Finally, although children were instructed to compare the angles between both Pacmen, they might solved the task instead by comparing the distance between both mouth sides. In other words, they compared length instead of angles. Since both dimensions depend on continuous spatial processing no differences are expected (please see also Fias et al., 2003). Moreover, many studies use the comparison of length to examine continuous spatial representation (de Hevia and Spelke, 2010; de Hevia et al., 2012b; Dormal and Pesenti, 2013). However, in the present study, children were instructed to compare angles and it can be assumed that the majority did pay attention to angles and not to line length. A further advantage of angle comparison is the similarity to dot comparison as both tasks need spatial processing in two-dimensions that comprise comparable spatial extent. In contrast, spatial line length elongation is smaller compared to the spatial extent of arrays of dots why angle comparison is favored in the present study.

Finally, to gain a clearer picture of the developmental trajectories of continuous and discrete magnitude processing, future studies should also investigate younger children and measure reaction times to obtain a finer and continuous dimension of performance levels. Moreover, it would be very interesting to relate reaction times or accuracy levels to individual basic numerical and mathematical skills. To do so, future studies should assess a wide range of basic numerical and mathematical abilities that rely more or less on visuo-spatial magnitude processing and relate these skills to individual continuous and discrete magnitude functions.

## Conclusion

Research has revealed a largely inconclusive picture with respect to the association between numerical and spatial magnitude processing and a common underlying general magnitude system. Our findings provide new insights about the relation of discrete non-symbolic number processing (comparison of dot arrays) and continuous spatial processing (comparison of angle sizes) in children from the third to the sixth grade. Specifically, our results suggest that continuous spatial and discrete number processing are related to each other, but that continuous spatial representations might develop earlier than discrete number representations and are easier to be compared for children at this age range. In conclusion, present findings favor the existence of a more complex underlying magnitude system consisting of dissociated but closely interacting parts for continuous and discrete magnitude processing.

## Author Contributions

KK and UM conceived and planned the experiments and tested all the children. KK prepared all the data, performed the statistical data analyses, and wrote the manuscript. UM, MvA, and RO’GT provided the critical feedback to the final version of the manuscript.

## Conflict of Interest Statement

The authors declare that the research was conducted in the absence of any commercial or financial relationships that could be construed as a potential conflict of interest.

## Abbreviations

M: mean
N: number
p: statistical *p*-value
r: Pearson correlation coefficient
SD: Standard Deviation
t: Student’s *t*-test value.
