# Supplementary material for: Development of a Possible General Magnitude System for Number and Space
Source: Front Psychol. 2018 Nov 19;9:2221. doi: 10.3389/fpsyg.2018.02221 (PMC6252337; doi:10.3389/fpsyg.2018.02221)
Supplement: Supplementary file 1 [file Table_1.DOC]

**Table S1:** **Spatial comparison task structure**

| **Trial** | **Pacman 1** | **Pacman 2** | | |
| --- | --- | --- | --- | --- |
|  |  | *Angle* | *Variation (%)* | *Ratio* |
| 1 | 45° | 18° | -60 | 0.40 |
| 2 | 45° | 40° | -10 | 0.89 |
| 3 | 45° | 59° | +30 | 0.76 |
| 4 | 45° | 23° | -50 | 0.51 |
| 5 | 45° | 32° | -30 | 0.71 |
| 6 | 45° | 42° | -5 | 0.93 |
| 7 | 45° | 47° | +5 | 0.96 |
| 8 | 45° | 63° | +40 | 0.71 |
| 9 | 45° | 68° | +50 | 0.66 |
| 10 | 45° | 27° | -40 | 0.60 |
| 11 | 45° | 59° | +30 | 0.76 |
| 12 | 45° | 18° | -60 | 0.40 |
| 13 | 45° | 54° | +20 | 0.83 |
| 14 | 45° | 42° | -5 | 0.93 |
| 15 | 45° | 47° | +5 | 0.96 |
| 16 | 45° | 23° | -50 | 0.51 |
| 17 | 45° | 49° | +10 | 0.92 |
| 18 | 45° | 36° | -20 | 0.80 |
| 19 | 45° | 54° | +20 | 0.83 |
| 20 | 45° | 72° | +60 | 0.63 |
| 21 | 45° | 32° | -30 | 0.71 |
| 22 | 45° | 63° | +40 | 0.71 |
| 23 | 45° | 27° | -40 | 0.60 |
| 24 | 45° | 49° | +10 | 0.92 |
| 25 | 45° | 36° | -20 | 0.80 |
| 26 | 45° | 40° | -10 | 0.89 |
| 27 | 45° | 72° | +60 | 0.63 |
| 28 | 45° | 68° | +50 | 0.66 |

The mouth angle of one Pacman was always 45 degrees and the mouth angle of the other Pacman varied between minimum 18 degrees to maximum 72 degrees (18, 23, 27, 32, 36, 40, 42, 47, 49, 54, 59, 63, 68, 72 degrees (2 trials for each degree)). Difficulty level was controlled by varying percentage variation of 45° (+/- 5°, +/- 10°, +/- 20°, +/- 30°, +/- 40°, +/- 50°, +/- 60° (2 trials for each variation)) and accordingly the ratio varied between both presented mouth angels across trials (0.40 to 0.96). In addition, the side of the correct answer and color of Pacmen were balanced.
