# Supplementary material for: Development of a Possible General Magnitude System for Number and Space
Source: Front Psychol. 2018 Nov 19;9:2221. doi: 10.3389/fpsyg.2018.02221 (PMC6252337; doi:10.3389/fpsyg.2018.02221)
Supplement: Supplementary file 2 [file Table_2.DOC]

**Table S2: Non-symbolic number comparison task structure**

| **Trial** | **Dot array 1** | **Dot array 2** | | |
| --- | --- | --- | --- | --- |
|  |  | *Number* | *Variation (%)* | *Ratio* |
| 1 | 20 | 32 | +60 | 0.63 |
| 2 | 20 | 12 | -40 | 0.60 |
| 3 | 20 | 28 | +40 | 0.71 |
| 4 | 20 | 24 | +20 | 0.83 |
| 5 | 20 | 26 | +30 | 0.77 |
| 6 | 20 | 22 | +10 | 0.91 |
| 7 | 20 | 10 | -50 | 0.50 |
| 8 | 20 | 26 | +30 | 0.77 |
| 9 | 20 | 18 | -10 | 0.90 |
| 10 | 20 | 28 | +40 | 0.71 |
| 11 | 20 | 12 | -40 | 0.60 |
| 12 | 20 | 8 | -60 | 0.40 |
| 13 | 20 | 8 | -60 | 0.40 |
| 14 | 20 | 22 | +10 | 0.91 |
| 15 | 20 | 14 | -30 | 0.70 |
| 16 | 20 | 26 | +30 | 0.77 |
| 17 | 20 | 10 | -50 | 0.50 |
| 18 | 20 | 14 | -30 | 0.70 |
| 19 | 20 | 16 | -20 | 0.80 |
| 20 | 20 | 30 | +50 | 0.67 |
| 21 | 20 | 32 | +60 | 0.63 |
| 22 | 20 | 24 | +20 | 0.83 |
| 23 | 20 | 18 | -10 | 0.90 |
| 24 | 20 | 14 | -30 | 0.70 |
| 25 | 20 | 28 | +40 | 0.71 |
| 26 | 20 | 30 | +50 | 0.67 |
| 27 | 20 | 16 | -20 | 0.80 |
| 28 | 20 | 12 | -40 | 0.60 |

The number of dots of one array was always 20 and the number of dots of the other array varied between minimum 8 to maximum 32 degrees (8, 10, 12, 14, 16, 18, 22, 24, 26, 28, 30, 32). Difficulty level was controlled by varying percentage variation of 20 dots (+/- 10°, +/- 20°, +/- 30°, +/- 40°, +/- 50°, +/- 60°) and accordingly the ratio varied between both presented sets of dots (0.40 – 0.91). In addition, the side of the correct answer and color of Pacmen were balanced.
