# Supplementary material for: Development of a Possible General Magnitude System for Number and Space
Source: Front Psychol. 2018 Nov 19;9:2221. doi: 10.3389/fpsyg.2018.02221 (PMC6252337; doi:10.3389/fpsyg.2018.02221)
Supplement: Supplementary file 3 [file Table_3.DOC]

**Table S3: Balanced ratios & mean accuracies**

| **Ratio** | **Dots** | | | **Angles** | | |
| --- | --- | --- | --- | --- | --- | --- |
|  | *Trial* | *Exact Ratio* | *Accuracy (%)* | *Trial* | *Exact Ratio* | *Accuracy (%)* |
| 0.40 | 12 | 0.40 | 100 | 12 | 0.40 | 100 |
|  | 13 | 0.40 | 100 | 1 | 0.40 | 99 |
| 0.50-0.51 | 17 | 0.50 | 100 | 16 | 0.51 | 100 |
|  | 7 | 0.50 | 100 | 4 | 0.51 | 99 |
| 0.60 | 28 | 0.60 | 100 | 10 | 0.60 | 100 |
|  | 11 | 0.60 | 100 | 23 | 0.60 | 99 |
|  | 2 | 0.60 | 99 |  |  |  |
| 0.63 | 1 | 0.63 | 99 | 20 | 0.63 | 99 |
|  | 21 | 0.63 | 98 | 27 | 0.63 | 99 |
| 0.66-0.67 | 20 | 0.67 | 99 | 28 | 0.66 | 99 |
|  | 26 | 0.67 | 98 | 9 | 0.66 | 100 |
| 0.71 | 10 | 0.71 | 99 | 21 | 0.71 | 97 |
|  | 3 | 0.71 | 97 | 5 | 0.71 | 99 |
|  | 25 | 0.71 | 95 | 8 | 0.71 | 98 |
|  |  |  |  | 22 | 0.71 | 96 |
| 0.76-0.77 | 16 | 0.77 | 96 | 11 | 0.76 | 98 |
|  | 5 | 0.77 | 97 | 3 | 0.76 | 97 |
|  | 8 | 0.77 | 97 |  |  |  |
| 0.80 | 27 | 0.80 | 95 | 18 | 0.80 | 94 |
|  | 19 | 0.80 | 97 | 25 | 0.80 | 95 |
| 0.83 | 4 | 0.83 | 94 | 13 | 0.83 | 99 |
|  | 22 | 0.83 | 82 | 19 | 0.83 | 87 |
| 0.89-0.90 | 23 | 0.90 | 83 | 2 | 0.89 | 94 |
|  | 9 | 0.90 | 84 | 26 | 0.89 | 87 |
| 0.91-0.92 | 6 | 0.91 | 63 | 17 | 0.92 | 91 |
|  | 14 | 0.91 | 64 | 24 | 0.92 | 86 |
| excluded | 18 | 0.70 | 98 | 6 | 0.93 | 41 |
|  | 24 | 0.70 | 98 | 14 | 0.93 | 80 |
|  | 15 | 0.70 | 98 | 7 | 0.96 | 72 |
|  |  |  |  | 15 | 0.96 | 90 |

Trial number (1-28), ratio between sets to be compared, and mean accuracy (%) is indicated for both task (dots and angles). Since difficulty levels are influenced by ratios between sets to be compared, we excluded the ratio 0.70 of dot comparisons and the ratios 0.93 and 0.96 of angle comparisons to balance ratios between both conditions. Whenever comparisons between both tasks were performed, only trials of balanced ratios were included. Also for the correlation between ratio and accuracy, only balanced ratios were included.
